# A novel splice variant of the protein tyrosine phosphatase *PTPRJ* that encodes for a soluble protein involved in angiogenesis

**DOI:** 10.18632/oncotarget.14350

**Published:** 2016-12-29

**Authors:** Anna Bilotta, Vincenzo Dattilo, Sabrina D'Agostino, Stefania Belviso, Stefania Scalise, Mariaconcetta Bilotta, Eugenio Gaudio, Francesco Paduano, Nicola Perrotti, Tullio Florio, Alfredo Fusco, Rodolfo Iuliano, Francesco Trapasso

**Affiliations:** ^1^ Department of Medicina Sperimentale e Clinica, University Magna Graecia of Catanzaro, Catanzaro, Italy; ^2^ Department of Scienze della Salute, University Magna Graecia of Catanzaro, Catanzaro, Italy; ^3^ Lymphoma and Genomics Research Program, Institute of Oncology Research (IOR), Bellinzona, Switzerland; ^4^ Tecnologica Research Institute, Biomedical Section, Crotone, Italy; ^5^ Laboratory of Pharmacology, Dept. of Internal Medicine, and Center of Excellence for Biomedical Research (CEBR), University of Genova, Genova, Italy; ^6^ Istituto di Endocrinologia e Oncologia Sperimentale - CNR c/o Dipartimento di Medicina Molecolare e Biotecnologie Mediche, University Federico II of Napoli, Napoli, Italy

**Keywords:** protein tyrosine phosphatase, soluble isoform, angiogenesis, glioblastoma, angiogenic factor

## Abstract

PTPRJ is a receptor protein tyrosine phosphatase with tumor suppressor activity. Very little is known about the role of PTPRJ ectodomain, although recently both physiological and synthetic PTPRJ ligands have been identified. A putative shorter spliced variant, coding for a 539 aa protein corresponding to the extracellular N-terminus of PTPRJ, is reported in several databases but, currently, no further information is available.

Here, we confirmed that the PTPRJ short isoform (named sPTPRJ) is a soluble protein secreted into the supernatant of both endothelial and tumor cells. Like PTPRJ, also sPTPRJ undergoes post-translational modifications such as glycosylation, as assessed by sPTPRJ immunoprecipitation. To characterize its functional activity, we performed an endothelial cell tube formation assay and a wound healing assay on HUVEC cells overexpressing sPTPRJ and we found that sPTPRJ has a proangiogenic activity. We also showed that sPTPRJ expression down-regulates endothelial adhesion molecules, that is a hallmark of proangiogenic activity. Moreover, *sPTPRJ* mRNA levels in human high-grade glioma, one of the most angiogenic tumors, are higher in tumor samples compared to controls. Further studies will be helpful not only to clarify the way sPTPRJ works but also to supply clues to circumvent its activity in cancer therapy.

## INTRODUCTION

The molecular signaling of the Protein Tyrosine Phosphatase Receptor J (PTPRJ) is implicated in a wide variety of physiological and pathological processes, and the level of this protein is important for modulation of the activity of various tyrosine kinase receptors [1–6]. While PTPRJ extracellular region, formed by nine fibronectin-like domains, is crucial for the binding of ligands, as Syndecan-2 and Thrombospondin-1 [7, 8], its intracellular region is composed of a single catalytic domain [9]. PTPRJ expression is decreased in many cancer cells [10, 11] and its restoration is able to suppress the malignant phenotype [12–14]. PTPRJ also shows a significant role in angiogenesis, since in endothelial cells, the dephosphorylation of Vascular Endothelial Growth Factor Receptor-2 (VEGFR-2) mediated by PTPRJ results in the inhibition of the Vascular Endothelial Growth Factor (VEGF)-induced mitogenic signals [5, 15, 16].

Besides the classical receptor-type phosphatase, *PTPRJ* encodes for an alternative spliced variant (NM_001098503.1 - NCBI Gene database) that includes the first eight exons of *PTPRJ* and an alternative exon nine with a stop-codon. The putative 539 aa protein coded by the small transcript is formed by the first five type III fibronectin domains of the extracellular region of PTPRJ and for this reason, it is predicted to be secreted by the cells. So far, no experimental evidence of the expression and function of the short form of PTPRJ (named sPTPRJ from herein) are reported.

Alternative splicing of messenger RNA during the maturation of pre-mRNA is an important post-transcriptional regulation mechanism in eukaryotic cells. A large number of genes in eukaryotes are alternatively spliced into several forms. In this way, multiple proteins with different functions can be produced from a single gene [17, 18]. Many soluble receptors are produced by alternative splicing, being the translation of receptor mRNA prematurely terminated and producing receptors that lack the transmembrane and cytoplasmic domains [19–22]. Protein-tyrosine phosphatases (PTPs) with different forms generated by alternative splicing that have equal, contrasting or different functions have been already described. Sakurai T. *et al*. identified proteins corresponding to three splicing variants of the protein tyrosine phosphatase RPTP beta. Two of these forms are receptors that differ in a large extracellular domain, the third one is a secreted proteoglycan called phosphacan and lacks the cytoplasmic phosphatase domains [23, 24]. In rodents, four different isoforms of PTPRG, a transmembrane protein that behaves as a tumor suppressor, are derived from alternative splicing. One of these, PTPRG-S, is shown to be an extracellular variant secreted into culture medium when expressed in COS7 cells and, recently, Moratti *et al*. have shown its expression in human and murine plasma and tissues [25–28].

In this study, for the first time, we identified and characterized sPTPRJ. We show that sPTPRJ is a glycosylated protein expressed in many cell lines and secreted into cell culture medium. It is able to promote angiogenesis and cell migration. Moreover, *sPTPRJ* mRNA levels are upregulated in high-grade glioma, a tumor in which elevated angiogenesis plays a fundamental role to confer high-grade malignancy and poor prognosis [29–33].

## RESULTS

### sPTPRJ protein is endogenously expressed in various cell lines

**Figure 1:**
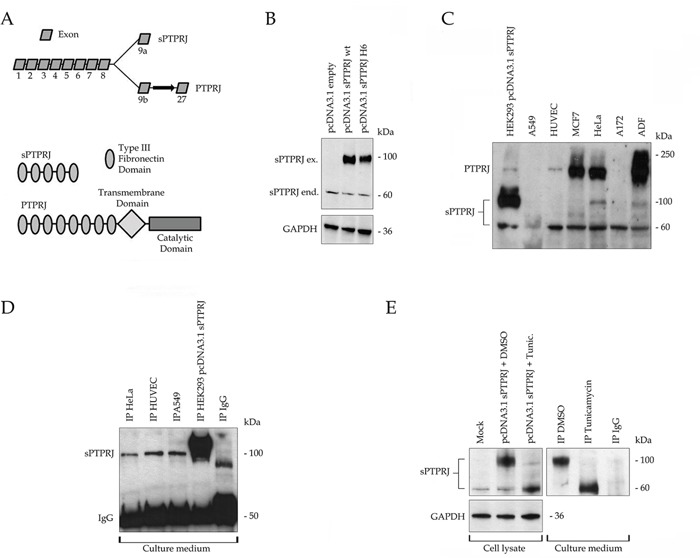
sPTPRJ is an endogenously expressed protein with extracellular localization and different glycosylation states **A.** The upper panel shows the two mRNA variants produced by alternative 9a/b exons; the spliced (3193bp) and the full length (7854bp) forms of PTPRJ. The bottom panel represents the structure of the relative codified proteins. In particular, the 539 aa sPTPRJ protein is composed of 5 type III fibronectin domains, instead of the 1337 aa PTPRJ protein is formed by 9 type III fibronectin extracellular domains, a transmembrane and a single catalytic cytoplasmic domain. **B.** The spliced mRNA variant codify for the sPTPRJ protein; HEK293 cells were transfected with pcDNA3.1 vector, empty or carrying sPTPRJ-WT/H6. Cell lysates were subjected to immunoblots using an anti-PTPRJ antibody specific for the extracellular portion of the protein. Two protein bands with different molecular weight are detectable, one at about 60 kDa and the other at about 100 kDa. Anti-GAPDH antibody was used to check equal loading. **C.** sPTPRJ is endogenously expressed in several normal and cancer cell lines. A549, HUVEC, MCF7, HeLa, A172 and ADF cell lysates were subjected to immunoblots using an anti-PTPRJ antibody specific for the extracellular portion of the protein. All cell lines express the 60 kDa protein, others, as HeLa cells, the 60 kDa and the 100 kDa forms. Lysate of HEK293 cells transfected with sPTPRJ/pcDNA3.1 was used as positive control. **D.** sPTPRJ is secreted in the extracellular compartment. Immunoprecipitation of sPTPRJ from cell culture media of HeLa, A549 and HUVEC cells was performed. Detection was carried out using an anti-PTPRJ antibody. Cell culture medium of sPTPRJ/pcDNA3.1 transfected HEK293 cells was used as positive control. **E.** sPTPRJ possesses different glycosylation states. HEK293 cells transfected with sPTPRJ/pcDNA3.1 were treated with 5 μg/ml Tunicamycin or DMSO as negative control. In the left panel, cell lysates were subjected to immunoblots using an anti-PTPRJ antibody specific for the extracellular portion of the protein. Anti-GAPDH antibody was used to check equal loading. In the right panel, immunoprecipitation of sPTPRJ from cell culture media was performed. Detection was carried out using an anti-PTPRJ antibody.

As previously described, the alternative splicing of *PTPRJ* mRNA provides a 3193bp transcript, named *sPTPRJ*, much smaller than *PTPRJ* transcript formed by 7854 bp. These transcripts differ in their 3′UTR (untranslated region) sequences; in particular, *PTPRJ* untranslated region has been well studied by Paduano *et al*. [34]; conversely *sPTPRJ* 3′UTR has not yet been studied. The 539 aa sPTPRJ protein is formed by five type III fibronectin domains whereas the 1337 aa PTPRJ protein is formed by nine type III fibronectin extracellular domains, a short transmembrane region and a catalytic cytoplasmic domain [9]. Transcripts (top panel) and proteins (bottom panel) are represented in Figure 1A.

First, the entire coding sequence of *sPTPRJ* was cloned into the pcDNA3.1 vector as described in Material and Methods. The expected molecular weight of sPTPRJ is approximately 60 kDa. However, two protein bands of approximately 100 kDa and 60 kDa were identified by Western blot in transfected HEK293 cells (Figure 1B). In order to understand which form of sPTPRJ was expressed in various cell lines, we performed a Western blot analysis. Most of the cell lines expressed only the 60 kDa form, whereas others (i.e., HeLa cells) expressed both forms. sPTPRJ was expressed either in adherent or suspension cell cultures (i.e., KASUMI cells), as well as in tumor and normal (i.e., HUVEC, Human Umbilical Vein Endothelial Cells) cell lines (Figure 1C). These results suggest that sPTPRJ, in a similar manner to PTPRJ [35], is an ubiquitously expressed protein.

### sPTPRJ is a soluble protein secreted in extracellular compartment

Since sPTPRJ does not possess a transmembrane domain, it should have an extracellular localization. To confirm this hypothesis, immunoprecipitation of sPTPRJ was performed from the culture medium of HeLa, HUVEC and A549 cells that possess different levels and forms of sPTPRJ (Figure 1D). Cell culture medium of s*PTPRJ*/pcDNA3.1 transfected HEK293 cells was used as positive control. Interestingly, only the 100 kDa form of sPTPRJ was immunoprecipitated from the culture medium of cell lines. The 60 kDa detected in the cell lysates (Figure 1C) did not immunoprecipitate from the culture medium. These data could mean that the sPTPRJ form with higher molecular weight is the only secreted form. However, since in some cell lines, the 100 kDa band observed in the immunoprecipitates from the medium is not detectable in cell lysates, the possibility of an unspecific precipitation of a 100 kDa protein could not be totally discarded. Alternatively, in some cell lines, the 100 kDa form of sPTPRJ could be synthesized and then rapidly secreted in the medium.

In some cell lines, expression of PTPRJ is increased in confluent cells in comparison to sparse cells [35]. To find if this happens also in the case of sPTPRJ, HeLa and ADF cells were grown and total RNA were then extracted from sparse (approximately 50 percent confluency) and confluent cells. In both cell lines the expression of *PTPRJ* increased in confluent cells, whereas no change was detected in *sPTPRJ* mRNA levels (Supplementary figure).

### sPTPRJ is endogenously N-glycosylated

The analysis of the protein sequence of sPTPRJ has identified twenty putative N-glycosylation sites (Uniprot database). On this basis, we hypothesized that the glycosylation state of the protein might be responsible for the two different molecular weight bands identified by Western blot (Figures 1B-1C). For this reason, HEK293 cells were transfected with *sPTPRJ*/pcDNA3.1 and then treated with Tunicamycin, a potent inhibitor of eukaryote N-acetylglucosamine transferases. Cells and culture media were collected and Western blot of lysates and immunoprecipitation from cell culture media were performed. Western blot of sPTPRJ in lysates of tunicamycin-treated cells revealed that the intensity of protein band of 60 kDa increased whereas the intensity of the 100 kDa sPTPRJ form decreased significantly in comparison with the control sample (Left panel Figure 1E). Moreover, immunoprecipitation from cell culture media showed that both forms of sPTPRJ could be secreted, indicating that N-glycosylation is not indispensable for protein secretion, but it could be needed to regulate the maturation of the protein (Right panel Figure 1E).

### sPTPRJ expression does not influence cell proliferation

**Figure 2:**
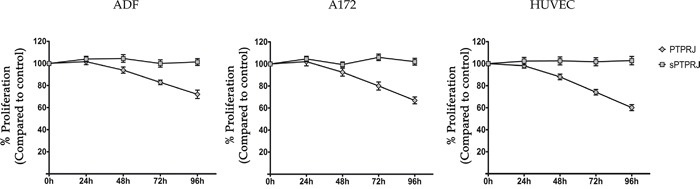
Cell proliferation is not influenced by sPTPRJ protein expression To analyze cell proliferation, the Alamar Blue assay was performed. Primary endothelial HUVEC, glioblastoma A172 and ADF cells (2.5×103/well) were seeded in a 96-well plate. After 24h cells were infected with Ad5-PTPRJ or Ad5-sPTPRJ viruses; Ad5-GFP infection was used as control. The same day, Alamar Blue stock solution was added. Absorbance at wavelengths 570 nm and 600 nm was determined after 0, 24, 48, 72 and 96h of Alamar Blue incubation. The experiment was performed in octuplicate and data are represented as mean ± SD.

PTPRJ agonist peptides, identified by Paduano *et al*., are able to bind and activate PTPRJ in its extracellular portion and to reduce proliferation of cancer cells [36, 37]. In order to understand if sPTPRJ expression can directly influence proliferation and if it could be a soluble protein able to bind membrane receptors and influence cell proliferation, we analyzed the cell proliferation of HUVEC cells and glioblastoma ADF and A172 cell lines infected with Ad5-GFP, Ad5-*sPTPRJ* (generation and testing of *sPTPRJ* recombinant adenovirus are reported in Materials and Methods) or Ad5-*PTPRJ* [36]. In agreement with the anti-proliferative effect of the rat PTPRJ observed in glioblastoma cell lines [38], the proliferation rate of Ad5-*PTPRJ* infected cells was reduced compared to Ad5-GFP infected cells; however, there was no change in the proliferation of cells overexpressing sPTPRJ (Ad5-*sPTPRJ*, Figure 2).

### sPTPRJ expression in HUVECs activates angiogenesis and cell migration

Several studies reported the importance of PTPRJ in angiogenesis [16, 37, 39–43]. To establish if sPTPRJ is involved in angiogenesis, we carried out two different *in vitro* assays using endothelial cells.

**Figure 3:**
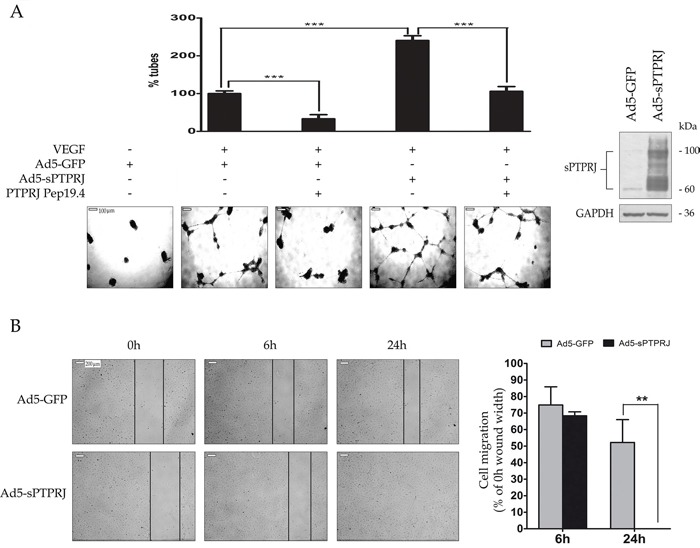
sPTPRJ protein increases tube formation and cell migration **A.** The angiogenic potential of sPTPRJ was determined by an endothelial cell tube formation assay performed on HUVEC cells infected with Ad5-sPTPRJ or Ad5-GFP as control. Cells (2.5×104/well) were plated on Matrigel and treated with VEGF and/or PTPRJ agonist peptide Pep19.4, as molecule able to inhibit tube formation. After 24h of incubation, capillary tube formation in each group was photographed using a light microscope. Representative photographs of tube formation or degeneration activity induced respectively by sPTPRJ or Pep19.4 and histograms with relative percentage are shown in the left panel. In the right panel, Western blot of the sPTPRJ expression was shown. Anti-GAPDH antibody was used to check equal loading. Results represent the mean ± SD of three independent experiments. One-way ANOVA followed by Bonferroni's multiple comparison test was used and P value was presented ≤ 0.001 =***. Scale bar = 100 μm. **B.** Wound healing assay was performed on HUVEC cells infected with Ad5-sPTPRJ or Ad5-GFP used as control. In the left panel, photographs show that cells overexpressing sPTPRJ, unlike control cells, are able to completely close the wound 24h after cells were scratched. In the right panel, cell migration is expressed as the percentage relative to 0h wound width. Data are presented as mean ± SD of three independent experiments and two-tailed Student's t-test was used. P values are presented as ≤ 0.01 =**. Scale bar = 200 μm.

HUVECs were infected with Ad5-GFP or Ad5-*sPTPRJ* and then seeded on a polymerized Matrigel layer, in the presence of VEGF. Kinetics of tube formation of endothelial cells were recorded as a proof of the initial step of neo-angiogenesis. Results showed that cells infected with the adenovirus expressing sPTPRJ are able to form tubes nearly three times more than control cells. To further investigate the role of sPTPRJ, we also treated cells with PTPRJ-19.4, a specific PTPRJ agonist peptide. In agreement with our recent published findings, the treatment of HUVEC cells with the PTPRJ agonist peptide [37] resulted in a reduction of new vessel formation (Figure 3A).

To further confirm the involvement of sPTPRJ in the endothelial cell migration, we performed a wound-healing assay. HUVEC cells were infected with empty adenovirus or adenovirus carrying *sPTPRJ*, and after 24h the cells were scratched. Photographs show that cells overexpressing sPTPRJ were able to completely close the wound in a time lower than 24h, whereas control cells did not (Figure 3B).

### sPTPRJ downregulates the expression of the endothelial adhesion molecules

**Figure 4:**
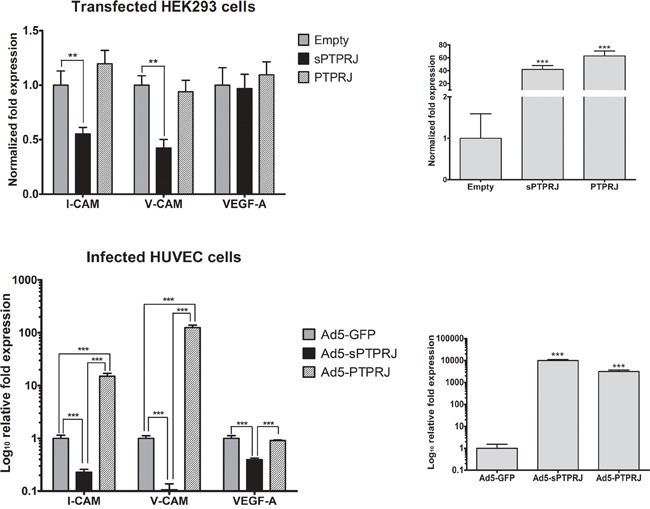
sPTPRJ overexpression affects I-CAM and V-CAM mRNA levels Real-time PCR analysis of I-CAM, V-CAM and VEGF-A was performed in sPTPRJ or PTPRJ transfected HEK293 (upper panel) and Ad5-sPTPRJ or Ad5-PTPRJ infected HUVEC cells (lower panel). PTPRJ and sPTPRJ overexpression were confirmed by real-time PCR (right panels). Values are plotted as fold change or Log10 fold change ± SD. One-way ANOVA followed by Bonferroni's multiple comparison test was used and P values are presented as ≤ 0.01 =**, ≤ 0.001 =***.

Angiogenic factors, such as bFGF (beta-fibroblast growth factor) and VEGF, are responsible for down-regulation of expression of the endothelial adhesion molecules ICAM-1 [44] and VCAM-1 [45]. To substantiate the potential role of sPTPRJ as an angiogenic factor, we analyzed the mRNA expression levels of *I-CAM* and *V-CAM* in *sPTPRJ*/pcDNA3.1 transfected HEK293 or Ad5-*sPTPRJ* infected HUVEC cells. Results showed that *I-CAM* and *V-CAM* mRNA levels decreased significantly compared with respective controls (Figure 4). Interestingly, the down-regulation of the adhesion molecules was not due to an increase of VEGF expression. The same experiment was carried out overexpressing PTPRJ, and in that case *I-CAM* and *V-CAM* mRNA levels increased significantly in infected HUVECs (Figure 4, lower panel).

### *sPTPRJ* mRNA levels are elevated in high-grade glioma samples

Glioblastoma, the highest grade of glioma, is a highly vascularized brain tumor and for its growth critically depends on the generation of tumor-associated blood vessels.

**Figure 5:**
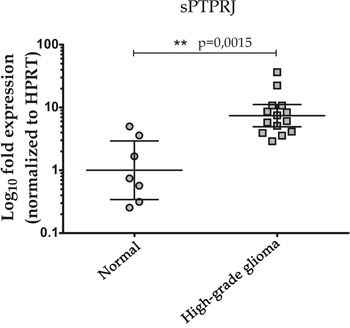
Increased mRNA levels of sPTPRJ in a high-grade glioma cohort Real-time PCR analysis of sPTPRJ was performed on brain tissue samples of non-tumorigenic [n=7] or high-grade glioma [n=14] subjects. Values were normalized to HPRT mRNA levels, represented as Log10 fold expression and plotted as geometric mean ± CI 95%. Mann-Whitney U-test was used.

In glioblastoma, high levels of vasculature have been reported to correlate with high-grade malignancy and poor prognosis [30]. To understand if sPTPRJ expression is changed in high-grade glioma tumor samples, we analyzed the mRNA expression levels of *sPTPRJ* in seven non-tumorigenic brain and fourteen high-grade glioma samples (four of grade III and ten of grade IV). Results showed significantly higher levels (sevenfold increase) of *sPTPRJ* mRNA in the glioma samples compared with controls (Figure 5).

## DISCUSSION

In this study, for the first time, we characterized the short variant of the receptor protein phosphatase PTPRJ generated by an alternative splicing. This protein, named sPTPRJ, contains only a portion of the N-terminal extracellular region of PTPRJ including the signal peptide, and for this reason it might be secreted into the extracellular space.

The identification of sPTPRJ cell compartmentalization is a first key step to understanding the possible mechanism of action. By experiments of immunoprecipitation and Western blot, we demonstrated that overexpressed sPTPRJ is secreted in the culture medium.

By Western blot, we detected two different protein isoforms corresponding to sPTPRJ and we showed that the diversity of molecular weight is due to changes in glycosylation, a post-translation modification that also occurs in the large form of PTPRJ [46].

Glycosylation is one of the most common protein post-translational modification. N-glycans can modulate several cancer cell mechanisms such as metabolism, signaling, growth, cell-cell adhesion, cell-matrix interaction, migration, invasion and metastasis [47]. Most secreted proteins, produced by alternative splicing, can be post-translationally glycosylated. For example, RPTPζ/phosphacan, the soluble spliced variant form of RPTP beta, is an important substrate for protein O-mannosylation in the brain [48].

Glycosylation of proteins could be important in the maturation and positioning of proteins [49]. In native conditions cell lines preferentially secreted the fully glycosylated sPTPRJ, but the treatment of transfected cells with tunicamycin does not block sPTPRJ secretion. Thus, N-glycosylation does not seem to affect the secretion of sPTPRJ but similarly to what occurs for other secreted proteins, it may be relevant to increase the protein stability [50–52].

Unlike *PTPRJ*, the expression of *sPTPRJ* did not show a density-dependent regulation. Since the two *PTPRJ* messenger RNAs share the same promoter but have different 3′ untranslated regions, the mechanism of density-dependent regulation, already observed for PTPRJ [35], could be connected to the 3′ untranslated region.

Since PTPRJ is involved in angiogenesis [5, 16, 37, 39–43], to investigate a possible functional role of sPTPRJ in this process, endothelial cell tube formation assay and wound healing assay were performed using HUVECs. Expression of sPTPRJ increases tube formation and endothelial cell migration, bringing evidence on its role as a *bona-fide* angiogenic factor.

Angiogenic factors, such as VEGF and bFGF, cause a down-regulation of endothelial adhesion molecules, such as ICAM-1 [44] and VCAM-1 [45], which is a hallmark of pro-angiogenic activity. Forced expression of sPTPRJ in HEK293 and HUVEC cells showed that *I-CAM* and *V-CAM* mRNA levels decreased significantly in both cell lines, differently *VEGF-A* mRNA levels did not change in HEK293 and significantly decreased in infected HUVECs. These data then support the role of sPTPRJ as an angiogenic factor. How to sPTPRJ can generate new vessels remains to be defined, but its effect seems to be not related to cell growth since its overexpression does not influence cell proliferation. Interestingly, in HUVECs a dramatic increase of *I-CAM* and *V-CAM* mRNA levels was detected after overexpression of PTPRJ. These results warrant the study of *PTPRJ* gene in endothelial response to inflammation.

Although the relevance of PTPRJ in normal blood circulation is clear [39], mechanisms that are involved in this process are not completely understood. In different studies, activation of PTPRJ resulted to inhibit angiogenesis through down-regulation of ERK activity [37, 40, 41] or enhance angiogenesis via SRC activation [16, 42, 43]. PTPRJ expression levels and different experimental models could justify these discrepancies, but the role of sPTPRJ has never been studied. Thus, sPTPRJ could be a new important player to take in consideration, able to influence pro-angiogenic or anti-angiogenic pathways regulated by PTPRJ.

The sPTPRJ protein might be a ligand for a transmembrane receptor or a decoy ligand for soluble proteins; it could then act on other proteins as a positive or negative regulator, operating on different interacting molecules.

For example, PTPRJ interacts with thrombospondin-1 [8] which is a pro-angiogenic protein [52] and the binding of PTPRJ to TSP-1 increases the catalytic activity of PTPRJ, which is also relevant in the angiogenesis inhibition. Moreover, the interaction between PTPRJ and TSP-1 occurs in the type 1 repeats of TSP-1, a fundamental protein region for the angiogenic role of TSP-1 [53]. Thus, sPTPRJ could also bind thrombospondin-1 interfering with TSP-1 binding to other ligands or alternatively could compete with the binding of PTPRJ reducing its tyrosine phosphatase catalytic activity.

The tumor vasculature of glioblastoma is characterized by a dense network of disorganized vessels with increased diameter and thickened basement membranes [30, 31]. Targeting angiogenesis has been and continues to be an attractive therapeutic modality in both newly diagnosed and recurrent glioblastoma patients [54]. Thus, glioblastoma is a good tumor model to test the possibility that sPTPRJ can contribute to tumor angiogenesis *in vivo*. The expression of *sPTPRJ* is significantly higher in high-grade glioma samples than in control brains, supporting that this protein could be involved in tumor angiogenesis.

Our data demonstrate that sPTPRJ can be considered a *bona-fide* angiogenic factor, and on the basis of these findings sPTPRJ may has a role in the feeding of glioblastoma cells by the generation of new tumor vessels. Taken together, these data show that sPTPRJ could represent a novel candidate biomarker in glioblastoma. More studies are needed to identify the interacting molecules and pathways involved in the sPTPRJ action and to study its expression in other cancer types.

## MATERIALS AND METHODS

### Cell lines and transfections

A549 lung cancer and ADF human glioblastoma cell lines were cultured in RPMI 1640 medium supplemented with 10% fetal bovine serum (Sigma-Aldrich) [55]. HeLa cervical cancer, MCF-7 mammary cancer, HEK293 human embryonic kidney and A172 human glioblastoma cell lines were maintained in DMEM medium supplemented with 10% FBS (Sigma-Aldrich). Human endothelial vein HUVEC cells were cultured in medium-200 supplemented with LSGS (Gibco). All cells were cultured in medium with 1% penicillin-streptomycin solution (Invitrogen) at 37°C in a humidified atmosphere with 5% CO_2_.

Transfections were performed in HEK293 cells. The day before transfection, cells were seeded at a density of 3×10^6^ in 60 mm plates. *sPTPRJ*-*WT/H6* and *PTPRJ* in pcDNA3.1 or pcDNA3.1 empty vector plasmids were mixed with Lipofectamine 2000 (Invitrogen) for 20 minutes at room temperature according to the manufacturer's instructions. The mixtures were then applied to the cells in a final volume of Opti-MEM I (Invitrogen). After incubation for 8 hours at 37°C, DMEM supplemented with serum was added. Cells were then cultured for additional 72 hours at 37°C before further analysis.

### Human samples

Human brain tumor samples were collected from the Neurosurgery Unit of the IRCCS-AOU San Martino IST (Genova, Italy), after patients’ informed consent and Institutional Ethical Committee approval. After the surgery, tumor specimens were immediately frozen at -80°C till the processing for mRNA extraction. Non-tumor brain samples derive from the Brain Bank at Case Western Reserve University (Cleveland, OH) and are a kind gift of Prof. Claudio Russo (University of Molise, Italy).

### Antibodies and Western blot analysis

Mouse monoclonal antibody against PTPRJ was purchased from R&D Systems. Rabbit polyclonal antibody against PTPRJ was purchased from Abcam. These antibodies were generated to recognize PTPRJ but they can also be used for sPTPRJ because are raised against the extracellular region of PTPRJ spanning from the first to the second fibronectin domain. Horseradish peroxidase(HRP)-conjugated anti-rabbit and anti-mouse, anti-GAPDH antibodies and normal mouse IgG were purchased from Santa Cruz Biotechnology.

Cells were lysed in lysis buffer (50 mM Tris-HCl pH 7.5, 150 mM NaCl, 0.5% NP-40, 1 mM Na_3_VO_4_, 0.1% aprotinin, 2 mM PMSF and 25 mM NaF) and incubated for 30 min on ice. Cellular debris were pelleted by centrifugation at 16.000g for 30 min at 4°C. Protein concentrations were determined using the Bradford protein assay dye (Bio-Rad). Total cell lysates were separated by SDS−PAGE and transferred to nitrocellulose membranes (Bio-Rad). Membranes were blocked in 5% nonfat dry milk (Bio-Rad) and then probed for 2 h with primary antibodies. After incubation with specific (HRP)-conjugated secondary antibodies, protein bands were revealed by the ECL detection system (Ge-Healthcare).

### Construction of *sPTPRJ* and *sPTPRJ-H6*/pcDNA3.1 vectors

A pBluescript-*PTPRJ* recombinant vector was digested with the Bpu1102I and XbaI restriction endonucleases. The 3′ region of *PTPRJ* from the Bpu1102I site until the stop codon of *PTPRJ* was removed and substituted with a Bpu1102I/XbaI fragment containing the 3′ of *sPTPRJ*. This fragment was generated by PCR amplification using Pfu DNA-polymerase, HeLa cDNA as template and the following primers: Forward (upstream of Bpu1102I site) 5′-GGTCAGCACGACGGAGA-3′; Reverse (*sPTPRJ*) 5′-CCATCTAGATCATCCAGTTCTATTGCAAACTGTC-3′; Reverse (*sPTPRJ-H6*) 5′-CCA TCTAGATCAGTGATGGTGATGGTGATGTCCAGTTCTATTGCAAACTGTC-3′. The pBluescript-*sPTPRJ* recombinant vectors were then digested with NotI and the full-length *sPTPRJ* were excised and sub-cloned into the pcDNA3.1 vector, linearized with the same restriction enzyme. Recombinant vectors were checked by sequencing.

### Immunoprecipitation from cell culture media and tunicamycin treatment

Cell culture media (10 ml) of HeLa, HUVEC, A549 and *sPTPRJ*/pcDNA3.1 transfected HEK293 cells (positive control) were collected and precleared with 15 μl of protein A/G plus-agarose(Santa Cruz Biotechnologies). Cell media were then incubated overnight with anti-PTPRJ or normal IgG antibodies and protein A/G plus-agarose. After five washes in PBS 1X, immunoprecipitated proteins were detached from the agarose beads by heat treatment at 99°C for 5 minutes in 2X loading buffer and then analyzed by Western blot.

To characterize the glycosylation state of sPTPRJ, HEK293 cells transfected with *sPTPRJ*/pcDNA3.1 were treated 24h after the transfection with 5 μg/ml Tunicamycin (Sigma-Aldrich) or DMSO (Sigma-Aldrich), as negative control. 48h later, cells were collected and Western blot of cell lysates and immunoprecipitated sPTPRJ from cell culture media was performed.

### Generation and validation of recombinant adenoviruses carrying a human *WT/H6*-tagged *sPTPRJ* cDNA

The newly generated *sPTPRJ* and *sPTPRJ-H6* cDNA were excided from *sPTPRJ* or *sPTPRJ-H6*/pCDNA3.1 with NotI and were cloned in the transfer vector pAdenoVator-CMV5(CuO)-IRES-GFP (Qbiogene) linearized with NotI. Recombinant transfer vectors were linearized with PmeI and then electroporated with the construct AdVator ΔE1/E3 containing the defective adenoviral genome into *E. coli* BJ5183 cells. The resulting vectors were linearized with PacI and transfected into HEK293 cells by Lipofectamine to package viruses. Single viral plaques were isolated, expanded, and checked for sPTPRJ or sPTPRJ-H6 expression. Viruses were purified by Vivapure AdenoPACK 100 (Vivascience) and titration was performed by the TCID50 method.

The adenoviruses carrying *sPTPRJ* or *sPTPRJ-H6* were validated by infection of A549 cells at a multiplicity of infection (MOI) of 25, analysis of Green Fluorescent Protein (GFP) by fluorescent microscopy (Leica), immunoblots of cell lysates and purification of His6-tagged protein from cell culture media by poly(His)-avid magnetic beads (Promega).

### Proliferation assay

The Alamar Blue assay (BioSource Int.) was used to measure cell proliferation. The dye used is an oxidation-reduction indicator and cellular metabolism induces a chemical reduction of the Alamar Blue medium. To perform the experiment, cells were infected with adenoviruses carrying *sPTPRJ* or *PTPRJ* and with an empty adenovirus as control. The appropriate MOI for cell line was used. Alamar Blue stock solution was aseptically added at 1:10 dilution. Absorbance at wavelengths 570 nm and 600 nm was determined by Multiskan GO (Thermo Scientific). Data were obtained following the manufacturer's protocols.

### Endothelial cell tube formation assay

Unpolymerized Matrigel (Becton Dickinson) diluted 1:2 in nude medium-200 was placed (50 μL/well) in a 96-well microtiter plate and polymerized for 1h at 37°C. HUVEC cells previously infected for 48h with adenovirus empty or carrying *sPTPRJ* at MOI 80, were seeded (2.5×10^4^/well) onto the solidified Matrigel in complete medium-200 added with VEGF (20 ng/ml) and/or PTPRJ agonist peptide pep19.4 at the concentration of 160 μM. After 24h of incubation at 37°C and 5% CO_2_, tube formation was analyzed by light microscopy (Leica). Quantification was performed by counting the number of tube-like structures in three randomly selected microscopic fields.

### Scratch assay

HUVEC cells were seeded in a 6-well plate to obtain after 72h a confluence of 100%. After 24h plated cells were infected at MOI 80 with adenovirus empty or carrying *sPTPRJ*. 48h later cell monolayers were scratched with a 0.2 ml pipette tip, washed twice, and photographs were taken. The width of wound at 6h and 24h was measured using Image J software (http://rsbweb.nih.gov/ij/). Cell migration was expressed as percentage relative to the width measured at the time zero.

### Quantitative real-time PCR

RNA extraction from *PTPRJ*/pcDNA3.1, s*PTPRJ*/pcDNA3.1 or pcDNA3.1 empty vector transfected HEK293 cells and from Ad5-*PTPRJ*, Ad5-*sPTPRJ* or Ad5-GFP infected HUVEC cells was performed with miRNeasy Mini Kit (Qiagen), following the manufacturer's protocol. Total RNA samples (1 μg) were subjected to the reaction of reverse-transcription using the High Capacity RNA-to-cDNA Kit (Applied Biosystems). One microliter of cDNAs was amplified by real-time PCR, in a BioRad IQ_5 apparatus, with Promega SYBR green kit and 10 pmol of primers in a total volume of 20 μl. The primers used to amplify endogenous *I-CAM1* mRNA were: 5′-ATGCCCAGACATCTGTGTCC-3′ (forward), 5′-GGGGTCTCTATGCCCAACAA-3′ (reverse). To amplify endogenous *V-CAM1* mRNA the primers were: 5′-GGGAAGATGGTCGTGATCCTT-3′ (forward), and 5′-TCTGGGGTGGTCTCGATTTTA-3′ (reverse). To amplify endogenous *VEGF-A* mRNA the primers were: 5′-CGAGGGCCTGGAGTGTGT-3′ (forward), and 5′-CGCATAATCTGCATGGTGATG-3′ (reverse).

RNAs from patients (fourteen high-grade glioma samples) and controls (seven non-tumorigenic brain samples) were extracted by trizol and reverse-transcribed as described above. Quantitative real-time PCR was performed to determine *sPTPRJ* mRNA levels. The primers used were: 5′-GTATTATCATTGGTGGCTTGTTC-3′ (forward) and 5′-AGGCAGGTGTTCAAATCATCC-3′ (reverse). Specific oligonucleotides used for amplification of hypoxanthine phosphoribosyl-transferase (*HPRT*) mRNA (normalization control) were reported by Vandesompele *et al*. [56]. Relative expression levels were calculated by the ΔΔCt method.

### Statistical methods and data analysis

Experiments were done at least three times, and results were expressed as mean ± standard deviation (SD). Proliferation assay was performed in octuplicate. Real-time PCR, tube formation and wound healing assays were performed in triplicate. Differences between groups were analyzed using unpaired two-tailed Student's *t*-test or one-way ANOVA followed by Bonferroni's multiple comparison test. To compare non-tumorigenic and high-grade glioma samples a Mann-Whitney *U*-test was used. Statistical analyses were performed using a statistics program (GraphPad Prism; GraphPad Software, La Jolla, CA, USA). In the figures, *P* value thresholds are represented as: *P* ≤ 0.05 =*, *P* ≤ 0.01 =** and *P* ≤ 0.001 =***.

## SUPPLEMENTARY MATERIALS FIGURES AND TABLES


