# Supplementary material for: A novel splice variant of the protein tyrosine phosphatase PTPRJ that encodes for a soluble protein involved in angiogenesis
Source: Oncotarget. 2016 Dec 29;8(6):10091–102. doi: 10.18632/oncotarget.14350 (PMC5354644; doi:10.18632/oncotarget.14350)
Supplement: Supplementary file 1 [file oncotarget-08-10091-s001.pdf]

A novel splice variant of the protein tyrosine phosphatase *PTPRJ* that encodes for a soluble protein involved in angiogenesis

SUPPLEMENTARY FIGURE

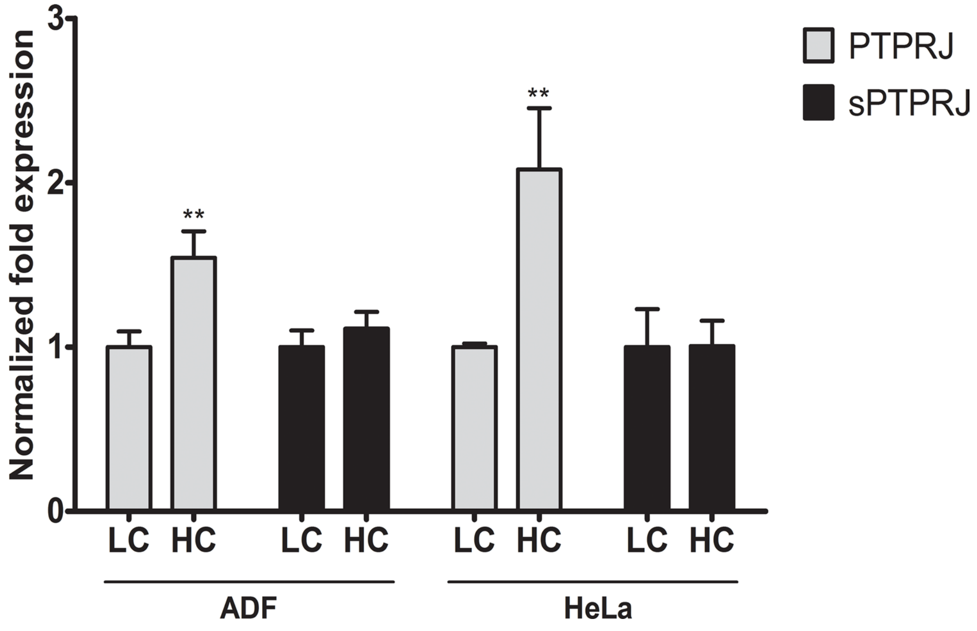

Supplementary Figure 1: *sPTPRJ* mRNA level is not influenced by confluency of cells. HeLa and ADF cells were grown and total RNA were then extracted from sparse (approximately 50 percent confluency) LC, and confluent cells, HC. Real-time PCR analysis of *PTPRJ* and *sPTPRJ* was performed and values were normalized to *HPRT* mRNA levels, represented as fold change ± SD. Differences between groups were analyzed using unpaired two-tailed Student's *t*-test and P value are presented as ≤ 0.01 =**.
